# Comparative Study of the Optical and Textural Properties of Tetrapyrrole Macrocycles Trapped Within ZrO_2_, TiO_2_, and SiO_2_ Translucent Xerogels

**DOI:** 10.3390/molecules201019463

**Published:** 2015-10-23

**Authors:** Eduardo Salas-Bañales, R. Iris Y. Quiroz-Segoviano, Luis Antonio Díaz-Alejo, Fernando Rojas-González, Alberto Estrella-González, Antonio Campero, Miguel A. García-Sánchez

**Affiliations:** Department of Chemistry, Universidad Autónoma Metropolitana-Iztapalapa, San Rafael Atlixco 186, Col. Vicentina, Mexico, D.F. 09340, Mexico; E-Mails: esalasb46@hotmail.com (E.S.-B.); irisyahel@hotmail.com (R.I.Y.Q.-S.); luis.alejo@msn.com (L.A.D.-A.); fernando@xanum.uam.mx (F.R.-G.); xanum.aeg@gmail.com (A.E.-G.); acc35@xanum.uam.mx (A.C.); mags@xanum.uam.mx (M.A.G.-S.)

**Keywords:** tetrapyrrolic macrocycles, porphyrin, phthalocyanine, sol-gel, ZrO_2_, TiO_2_, SiO_2_, hybrid materials

## Abstract

The entrapping of physicochemical active molecules inside mesoporous networks is an appealing field of research due to the myriad of potential applications in optics, photocatalysis, chemical sensing, and medicine. One of the most important reasons for this success is the possibility of optimizing the properties that a free active species displays in solution but now trapped inside a solid substrate. Additionally it is possible to modulate the textural characteristics of substrates, such as pore size, specific surface area, polarity and chemical affinity of the surface, toward the physical or chemical adhesion of a variety of adsorbates. In the present document, two kinds of non-silicon metal alkoxides, Zr and Ti, are employed to prepare xerogels containing entrapped tetrapyrrolic species that could be inserted beforehand in analogue silica systems. The main goal is to develop efficient methods for trapping or binding tetrapyrrole macrocycles inside TiO_2_ and ZrO_2_ xerogels, while comparing the properties of these systems against those of the SiO_2_ analogues. Once the optimal synthesis conditions for obtaining translucent monolithic xerogels of ZrO_2_ and TiO_2_ networks were determined, it was confirmed that these substrates allowed the entrapment, in monomeric form, of macrocycles that commonly appear as aggregates within the SiO_2_ network. From these experiments, it could be determined that the average pore diameters, specific surface areas, and water sorption capacities depicted by each one of these substrates, are a consequence of their own nature combined with the particular structure of the entrapped tetrapyrrole macrocycle. Furthermore, the establishment of covalent bonds between the intruding species and the pore walls leads to the obtainment of very similar pore sizes in the three different metal oxide (Ti, Zr, and Si) substrates as a consequence of the templating effect of the encapsulated species.

## 1. Introduction

During the last decades of the 20th century, the sol-gel process emerged as a new and interesting option for synthesizing and producing new types of hybrid materials [1]. The sol-gel procedure makes possible either the entrapment or the surface chemical bonding of many diverse active molecules, which range from simple cations [2] or small organic compounds [3] to complex biochemical species [4]. The keys of this versatility are: (i) the use of very reactive organometallic precursors, commonly metal alkoxides, to create a metal oxide pore network through hydrolysis and polycondensation sol-gel reactions, and (ii), the control of such reactions according to the nature and desired characteristics of the final solid system. The development and existence of new functionalized or modified alkoxides of varied metallic elements make possible the creation of a new kind of interesting and useful hybrid materials. Due to the relatively accessible and controllable thermal and chemical conditions under which the sol-gel process proceeds, this procedure is also known as “*chemie deuce”* or soft chemistry [1,5] and constitutes an important option for preserving the physicochemical properties of organic or biochemical molecules, which because of its own nature, cannot be trapped inside pore networks through the traditional impregnation or diffusion methods, which usually render lowly concentrated and heterogeneous systems.

**Figure 1:**
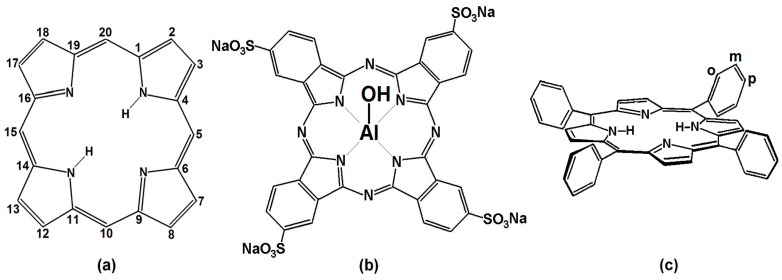
Chemical structures of: (**a**) free bases of porphyrin macrocycles; (**b**) the μ-*oxo-*tetrasulfophthalocyanine of Aluminum (III), (OH)AlTSPc; (**c**) *ortho-*, *meta-* and *para*-positions in *meso*-tetraphenylporphyrin (H_2_T(*o-*, *m-*, *p-S*)PP).

On the other hand, chlorophylls, the blood heme group and cytochromes [6,7,8] are natural tetrapyrrole macrocycles that perform transcendental functions in Nature. Synthetic analogues, such as porphyrins (H_2_P), phthalocyanines (H_2_Pc) (Figure 1), and naphthalocyanines (H_2_Nc), also display very important physicochemical properties. The synthesis of synthetic porphyrins could be achieved after the elucidation of the structures of chlorophyll, and hemoglobin’s heme group [9]. In all these centrosymmetric macrocyclic compounds, the π-electron system confers upon them high chemical and thermal stabilities, as well as other interesting properties that have led to a great number of applications [10,11,12,13,14,15,16].

Porphyrins are compounds derived from porphin which basic structure (Figure 1a), is constituted by four pyrrole rings bonded through methine groups (=CH), thus constituting an extensively conjugated double bond system. Subtraction of the central acidic hydrogens of these structures makes possible the creation of complexes with practically all metallic elements [7,8]. The phthalocyanines have structures related to porphyrin, but formed by four isoindole groups bonded by aza nitrogens and forming an extensive planar macrocycle with high chemical [17] and thermal stabilities, among other outstanding physicochemical properties [18], which result advantageous in catalysis [19], optics [20], optoelectronics [21], solar cells [22], and medicine [23,24]. Similarly, porphyrins and their metallic complexes show diverse and interesting properties that allow their application in similar fields [14,15,25,26,27,28,29,30]. Many times, in order to display the mentioned properties, macrocycles need to be trapped or fixed inside adequate solid porous substrates, such as carbon [31], hydrotalcites [19], zeolites [13], and metal oxide [16,26,27,28,29,30,32] or organic polymer networks [33].

For this reason, our research group has combined the transcendental physicochemical properties of synthetic and natural tetrapyrrolic macrocycles with the advantages provided by the sol-gel process to create hybrid materials with optimized properties. At the beginning of our research tetraethoxysilane (Si(OC_2_H_5_)_4_ = TEOS) was employed as the silica xerogel precursor and μ*-*hydroxyaluminum tetrasulphophthalocyanine, (OH)AlTSPc (Figure 1b), was chosen as an insertion probe in view of its good water solubility, thermal and chemical stability, low propensity for aggregation, and stability of its UV-Vis and fluorescence spectra along a wide pH range. In this way, it was established that a molar mixture sequence of TEOS:H_2_O:HCl:(OH)AlTSPc equivalent to 1:19.6:10^−3^:10^−3^–10^−7^ allowed the synthesis of translucent and monolithic silica xerogels, in which the macrocyclic species remained trapped in monomeric and chemically stable forms [31]. When using the same molar ratios, the physical trapping, in stable and monomeric form, of complexes of metal tetrasulphthalocyanines, MTSPc, [34], substituted tetraphenyl porphyrin free bases, H_2_T(*S*)PP, (Figure 1c) [35], as well as a series of miscellaneous porphyrinic complexes [36], within the pore structure of translucent and monolithic silica xerogels possible.

However, it was found for these systems that the interactions of the surface groups of the silica network, principally Si-OH surface species, and the trapped macrocycle molecule, hinder the efficient display of its physicochemical, and specially its spectroscopic properties [35,36]. In order to diminish this detrimental effect, some of the following strategies have been proposed and explored [35,37]: (i) to move as far as possible the macrocycle species from the walls of the pore network by establishing long covalent unions, between the tetrapyrrole macrocycle and the pore walls; (ii) to exchange the Si-OH groups by *alkyl* or aryl groups and; (iii) to trap the macrocycle inside non-siliceous networks, such as for instance ZrO_2_, TiO_2_, and Al_2_O_3_.

For placing the macrocyclic species far from the pore walls, it is necessary to establish bonds between the peripheral substituents of the macrocycle and the appropriate functional surface groups of the network precursors, such as silicon functionalized alkoxides (FA). By applying this strategy, the synthesized systems containing macrocyclic species covalently bonded to the pore walls of a silica network preserve better the fluorescence that is displayed in solution by the free macrocyclic species [38]. On the other hand, substitution of Si-OH groups by alkyl or aryl groups, arising from organo-substituted silicon alkoxides (OSA), induces an even better display of luminescent properties of the fixed macrocycle. Additionally, the intensities of the UV-vis and fluorescence spectroscopic signals of the trapped macrocycle depend on the identity and size of the alkyl or aryl groups attached to the pore walls [39,40]. Through this combined trapping strategy, it has been possible to optimize the fluorescence of synthetic tetrapyrrolic species, as for instance the free bases of *ortho-*, *meta-* or *para*-substituted *meso*-tetraphenylporphyrins (H_2_T(*o-*, *m- or p-S*)PP) (Figure 1c), as well as the coordination and fluorescence of natural tetrapyrrolic species, such as chlorophyll a [41]. Through the exploration of this second strategy, it was found that the attachment of organic chains induces a low polarity environment inside the pores, something that facilitates the occurrence of electronic transitions in the trapped macrocycles. In view of the success of the first two strategies for the encapsulation of tetrapyrrolic species within xerogel networks, in the present manuscript, we advance characterization results of hybrid materials containing macrocyclic species trapped or bonded inside inorganic sol-gel networks other than silica, *i.e.*, ZrO_2_ or TiO_2_ xerogels.

It is well known that zirconium oxide possesses great hardness, high fusion point, a thermal conductivity lower than that of common metals (2.09 W·m^−1^·K^−1^), a high refraction index of 2.21 (with λ = 630 nm), and a narrow band gap (3.8 to 3.2 eV). Consequently, zirconium oxide represents an interesting option for developing thin films, sensors, photoreactive materials, protective barriers, and optical devices such as waveguide materials [42].

Titanium oxide is a very important compound due to the semiconducting nature of its anatase crystal type, which is basic for photocatalytic systems [43] due to the low band gap energy (3.0 eV) of its rutile phase [44,45]. The different TiO_2_ phases are accessible and low cost if created through convenient preparation procedures [46,47,48,49]. Besides, these TiO_2_ materials are chemically and mechanically stable, while presenting a non-toxic character and being biocompatible with the environment and human tissues. Due to these properties, titanium oxide systems have been proposed to carry out the decomposition of organic substances, such as dioxins, phenols, aldehydes, aromatic compounds, herbicides and pesticides, as well as of inorganic compounds such as cyanides, nitriles, and poisonous compounds including heavy metals as for instance As, Pb, and Hg. In the same sense, TiO_2_ can be used to decompose sulphur and nitrogen oxides, eliminate odorous compounds such as ammonia, hydrogen sulphide, acetaldehyde, *etc.* Recently, TiO_2_ has been proposed as a self-cleaner in construction materials [50], since it promotes the inactivation or destruction of bacteria, protozoa, fungi, and virus. However, one of its most interesting applications is concerned with solar energy conversion, energy storage, green chemistry [51], and the synthesis of nanomaterials [52].

Due to all the above characteristics, in this contribution we will explore the possibility of synthesizing hybrid materials containing physically trapped or chemically bonded tetrapyrrolic macrocycles inside non-siliceous networks, created through the sol-gel method. Additionally, a comparative study of three different (Si, Ti and Or oxide) systems, encapsulating the same tetrapyrrolic macrocycles will be presented. For this reason, the present document reports results about the physical entrapment or the covalent bonding of macrocyclic species inside non-siliceous (Ti and Zr) networks. The macrocyclic compounds employed herein were selected on the basis of their previous successful incorporation inside silica systems [32,34,35,36,37,38,39,40]. These compounds include MTSPc species and a cobalt tetra-*para-*carboxyphenylporphyrin (CoT(*p-*COOH)PP) compound. Comparative analyses were performed to explore the applicability of the sol-gel method to synthesizing tetrapyrrolic macrocycle-based systems. Furthermore, it is important to ascertain if there exist or not important interactions and effects between the physically trapped or covalently bonded species and the surface groups of the pore network, and to explore if the intrinsic nature of the xerogel network affects the physicochemical stability and the aggregation propensity of the trapped species. In the end, our investigation will attempt to propose an explanation with respect to the observed differences existing among the three systems and most importantly, to suggest the best substrates to use for preserving the properties of different chemical species when trapped inside xerogel networks, and not only those related to tetrapyrrolic macrocycles.

## 2. Results and Discussion

### 2.1. UV-Vis Spectra of ZrO_2_ and TiO_2_ Systems

Because of its interesting and steady spectroscopic properties, the (OH)AlTSPc species (Figure 1) was chosen as a spectroscopic tracer to produce and optimize the molar composition of the precursor gellant mixture required to synthesize monolithic, translucent xerogels, in which these species can remain trapped in stable and monomeric forms. Similarly, a 4.04 × 10^−4^ M water solution of the (OH) AlTSPc species was used to determine the optimal experimental conditions required to synthesize monolithic and translucent ZrO_2_ and TiO_2_ xerogels, having tetrapyrrole macrocyclic species encapsulated in disaggregated and stable forms. Through this procedure, it was found that an Zr(OPr^n^)_4_:H_2_O:*n-*PrOH:acac molar ratio of 2:2.5:7.5:1, was the most adequate for creating the desired ZrO_2_ xerogels [53], while a 5.3:5:7:1 Ti(OPr^n^)_4_:H_2_O:*n-*PrOH:acac molar mixture was required for obtaining TiO_2_ xerogels. As it was found, the addition of some acetylacetone (acac) is necessary to lower the rates of the hydrolysis and polycondensation reactions, through the formation of the respective metallic acetylacetonate intermediates (M(OPrn)_3_·acac adduct species), which allowed the formation of translucent xerogel pore networks with the macrocyclic species trapped in stable and monomeric forms. This last supposition was verified by monitoring the UV-Vis spectrum of the (OH)AlTSPc molecules during all the sol-gel synthesis process until the final ZrO_2_ pore network was achieved.

**Figure 2:**
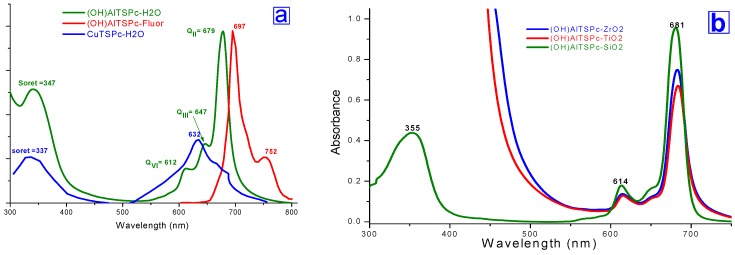
(**a**) UV-Vis spectra of monomeric (OH)AlTSPc species and CuTSPc aggregates of both in aqueous solution; and (**b**) UV-Vis spectra of (OH)AlTSPc species physically trapped inside ZrO_2_ and TiO_2_ pore networks.

As an example, the typical UV-Vis spectrum of the (OH)AlTSPc complex (Figure 2a) displays a Soret band (B) at about 347 nm and a more intense QII band at around 679 nm, which can be assigned to both a_2u_(π)→e_g_(π*) and a_1u_(π)→e_g_(π*)transitions, respectively [18]. Additionally, the Q_III_ band (appearing at 647 nm for the (OH)AlTSPc species) can be associated to the formation of cofacial phthalocyanine dimers, while a weak Q_IV_ satellite band of vibrational nature appears around 612 nm. Furthermore, the (OH)AlTSPc species displays an intense red fluorescence that constitutes an additional property useful to understand the physicochemical situation of the compound inside the pores of the xerogel network. Phthalocyanines are compounds that exhibit a high tendency to form aggregates; when phthalocyanine molecules form dimers or larger size aggregates, the respective UV-Vis spectrum only displays two bands, as in the case of copper tetrasulfophthalocyanine (CuTSPc), which shows a B band at around 337 nm and a QII band at 632 nm.

The UV-vis spectra of translucent ZrO_2_ and TiO_2_ xerogels containing the OH)AlTSPc species show bands at 667 and 602 nm for the ZrO_2_ sample and at 683 and 616 nm for the TiO_2_ specimen (Figure 2b). The Soret bands of phthalocyanine were masked by the intense band due to the TiO_2_ xerogel, which appears at wavelengths below 500 nm Nevertheless, in any case, the Q_I_ and Q_IV_ band pathway can be associated to the existence of monomers trapped inside the pore network. However, there exist slight wavelength position differences of these signals with respect to those observed in the spectrum corresponding to the (OH)AlTSPc species in solution, which can be mainly due to the different physicochemical environment existing inside either the ZrO_2_ or TiO_2_ networks.

The mixture molar composition, determined by means of the probing function of the (OH)AlTSPc species, suggests that it is possible to trap species similar to the last one, in monomeric and stable forms, inside the pores of ZrO_2_ and TiO_2_ networks. Consequently, the next step of the research consisted in trying to entrap metal tetrasulfopthalocyanine complexes (MTSPc) of cations such as Fe, Co, Ni and Cu. To achieve this goal, the respective MTSPc complex was first dissolved in the necessary amount of water, included in the corresponding molar mixtures of Zr(OPr^n^)_4_ or Ti(OPr^n^)_4_:H_2_O:*n-*PrOH:acac, which render a MTSPc concentration of 1.75 × 10^−3^ M. Additionally, 0.3 mL of DMF was added to the precursor mixtures of TiO_2_ xerogels or 0.083 mL in the case of the ZrO_2_ networks, as an aggregation inhibiting agent, something that was proved in experiments involving the (OH)AlTSPc probe species, since the materials obtained were rigid monoliths.

**Figure 3:**
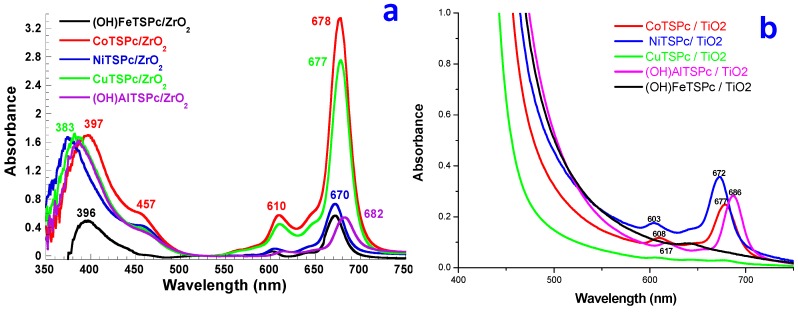
UV-vis spectra of final (**a**) ZrO_2_ and (**b**) TiO_2_ monolithic xerogel pore networks (using blank-ZrO_2_ or blank-TiO_2_ as background) containing encapsulated MTSPc species (M≡Fe, Co, Ni, Cu and Al).

The UV-Vis spectra of both ZrO_2_ and TiO_2_ xerogels (Figure 3) show Soret bands at around 380 to 400 nm, together with Q_IV_ and Q_II_ bands appearing at around 610 and 670–690 nm. These sets of signals mainly correspond to monomeric and stable structures of the MTSPc species. Since all samples were synthesized by employing the same MTSPc concentration, the intensity of the Q_II_ band depends only on the identity of the cation present in the MTSPc compound. An interesting result, in relation to these systems, is that the CuTSPc species, a complex having a high tendency to form aggregates, remained physically trapped in stable and monomeric forms inside the pores of ZrO_2_ networks, something that cannot be attained with TiO_2_ or with analogous silica samples [32,34]. Interestingly, dimers of the (OH)FeTSPc species remained trapped inside TiO_2_ networks and as, monomers inside silica networks [34].

The last results suggest that the MTSPc species remains immersed inside a more suitable physicochemical environment induced by the ZrO_2_ network than inside those generated by the TiO_2_ or SiO_2_ networks. This observation suggests that the ZrO_2_ matrix creates around the MTSPc species, a less polar environment than the two other substrates, which inhibits the tendency of the macrocycle to form aggregates thus facilitating the permanence of monomeric entrapped species throughout the sol-gel process and, consequently, in the final xerogels. This evidence could be associated to the weaker hydrophilic nature of ZrO_2_ together with the presence of a smaller population of M-OH surface groups, if compared to the cases of TiO_2_ or SiO_2_ networks created through the same procedure. The low polarity arises from the higher reactivity of Zr sand Ti alkoxide precursors, if compared to Si, something that produces a more condensed xerogel network and, consequently, a lower amount of remnant M-OH groups.

### 2.2. NIR Rehydration Monitoring of: (a) SiO_2_, (b) ZrO_2_, and (c) TiO_2_ Xerogels

**Figure 4:**
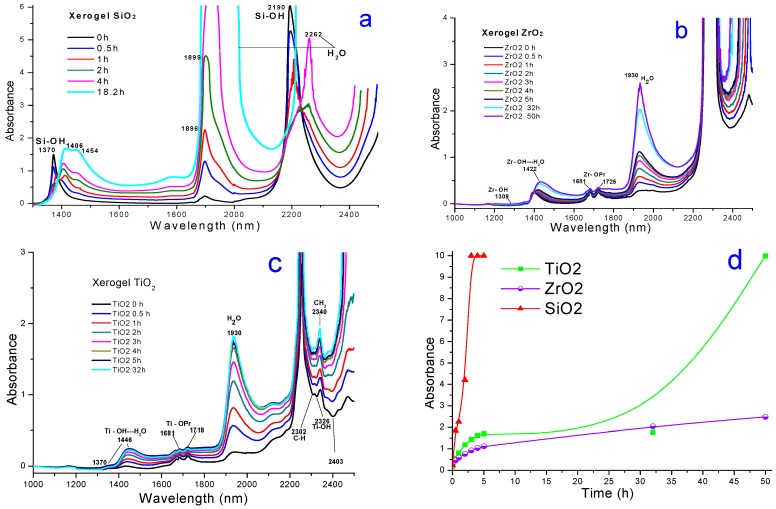
NIR monitoring of: (**a**) SiO_2_; (**b**) ZrO_2_; and (**c**) TiO_2_ xerogels during water rehydration at 125 °C; (**d**) The intensity of the bands associated to physisorbed water is plotted as function of the rehydration time.

With these hypotheses in mind, the rehydration of pristine SiO_2_, ZrO_2_ and TiO_2_ sol-gel substrates, was monitored through near infrared spectroscopy (NIR). The pristine SiO_2_ translucent xerogel was synthesized from a 1:19.6:10^−3^ molar mixture of TEOS:H_2_O:HCl and dried 24 h at 125 °C; afterwards, the SiO_2_ rehydration process was followed by NIR spectroscopy. Previous NIR studies on silica, synthesized by the sol-gel method and dehydrated at 800 °C (Figure 4a), showed signals of surface free or adjacent silanol (Si-O) groups at 2669 nm (ν_1_) and 2732 nm (ν_2_) [54]. When this sample was partially rehydrated, signals at 2817 nm (ν_3_) and a very wide band at 2920 nm (ν_4_) were observed; these signals are associated to the interaction of Si-OH groups with water [55,56]. The drastic effect of these interactions complicated the observation and monitoring of these fundamental vibrations; nevertheless, the analysis of overtones in the region extending from 1200 to 2500 nm can be more convenient to analyze.

In the respective NIR spectra of the dried SiO_2_ xerogel synthesized in this work (Figure 4a), band at 1370 nm can be observed, assigned to the first overtone (2ν_2_) of adjacent Si-OH groups, while the band at 2190 nm can be attributed to SiOH vibrations combined with the outer plane flexion of the water molecule ((ν_2_ + ν_OH_(flexion)) [57]. Finally, the band at 1896 nm can be associated to physisorbed water [58,59]. A dramatic increase in the intensity of the last signal (1899 nm) is evident when the xerogel is rehydrated; the remaining signals being replaced by new signals at 2262 (ν_3_+ ν_OH_(flexion)), 1454 (2ν_4_), and 1404 nm (2ν_3_) which are linked to Si-OH groups interacting with water. The monitoring of the process was stopped after 18.2 h, once the water signal masked the other bands appearing in the range 1900 to 2500 nm.

Similarly, the dried ZrO_2_ xerogel displayed signals at 1390 and 2270 nm associated to Zr-OH group vibrational overtones, while the signal at 1930 nm was attributed to physisorbed water. When the xerogel was rehydrated, the band at 1930 nm increased in intensity and new bands appeared at 1422 and 2300 nm, which can be related to Zr-OH groups interacting with physisorbed water. Furthermore, the bands in the range from 1681 to 1725 nm were attributed to C-H vibrations and associated to remnant alkoxide groups or acetylacetone. However, the increment in the intensity of the band associated to physisorbed water only remained stable after 50 h.

Finally, in the case of the dried TiO_2_ xerogel, the NIR spectrum exhibited a band at 2200 nm and another shallow band at 1370 nm, which can be associated to the vibrational overtones of Ti-OH groups; in turn, the band at 2340 nm can be linked to Ti-OH groups interacting with water. A weak band corresponding to physisorbed water can be observed at 1930 nm; however, the intensity of this signal increased and remained constant after 32 h as consequence of water readsorption. Additionally, the band at 2200 nm developed a higher intensity and the merging of a new strong band centered at 1446 nm masked the shallow band observed at 1370 nm. In turn, the bands located at 1681 and 1718 nm could be associated to remnant acetylacetone or alkoxide groups.

In Figure 4d the intensities of the bands linked to physisorbed water were plotted as a function of time for the three systems under analysis. In this graph, the rapid rehydration of the silica xerogel when compared to the same process occurring in TiO_2_ and ZrO_2_ xerogels is evident; this suggests a poorly accessible pore system or a lower hydrophilic character of these last two networks. The latter supposition can be associated to the existence of a lower population of Ti-OH or Zr-OH groups in the final xerogels, as a consequence of their higher reactivity, something that induces a more complete polycondensation of the hydrolyzed precursors. For example, the hydrolysis reaction times of zirconium alkoxides are of the order of microseconds, and are 105–108 times faster than their silicon alkoxide analogues [60].

In other words, there exist differences in the polarity existing inside the pore cavities of the SiO_2_, ZrO_2_, and TiO_2_ networks, which can be associated to the intrinsic nature of the inorganic network, but principally to the existence of different M-OH group populations. In the particular case of the MTSPc species, these differences may be responsible for the stability against aggregation of the macrocyclic species trapped inside the pores of each one of these metal oxide substrates.

The NIR spectra of the final ZrO_2_ or TiO_2_ xerogels encapsulating MTSPc species and dried at 125 °C, showed similar signals to those observed in the pristine networks. Interestingly, after similar rehydration times, the absorbance intensity of these bands at around 1900 nm, is associated to the amount of physisorbed water, obeying a sequence that depends on the macrocyclic species trapped inside the network pores. For instance, the ZrO_2_ xerogels followed the following decreasing intensity sequence: FeTSPc > CoTSPc > NiTSPc > CuTSPc > (OH)AlTSPc, which suggests that the amount of physisorbed water also depends on the identity of the metal cation that is present in the MTSPc complex. This interesting result suggests that the capacity of readsorption of water, and possibly, of other chemical species could be a function of the identity of the cation existing in the trapped MTSPc species inside the network pores.

### 2.3. TEM Images and SEM Mapping of ZrO_2_ and TiO_2_ Xerogels

**Figure 5:**
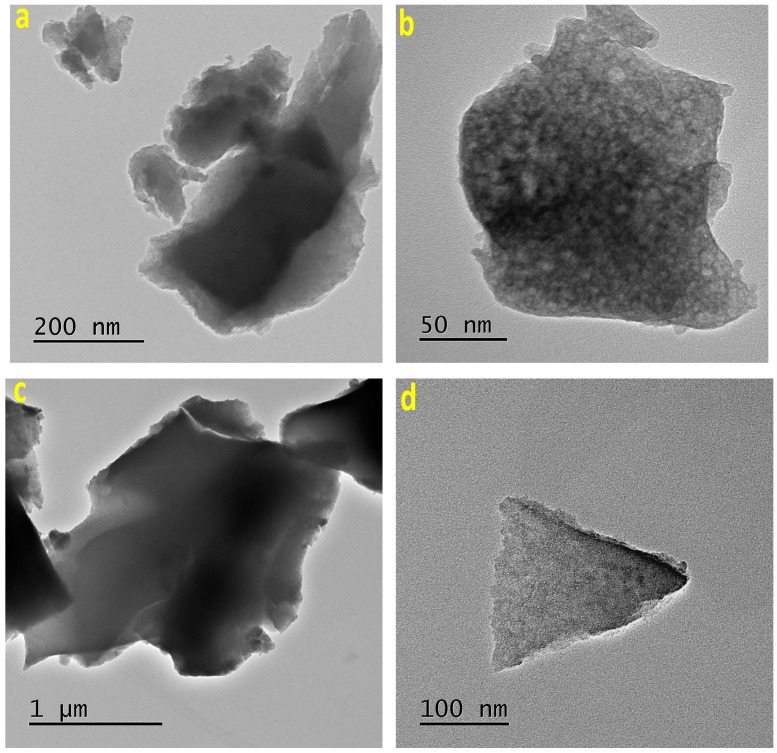
TEM images of a xerogel containing the (OH)AlTSPc species trapped within the pores of ZrO_2_ (**a**,**b**) and TiO_2_ (**c**,**d**) networks.

**Figure 6:**
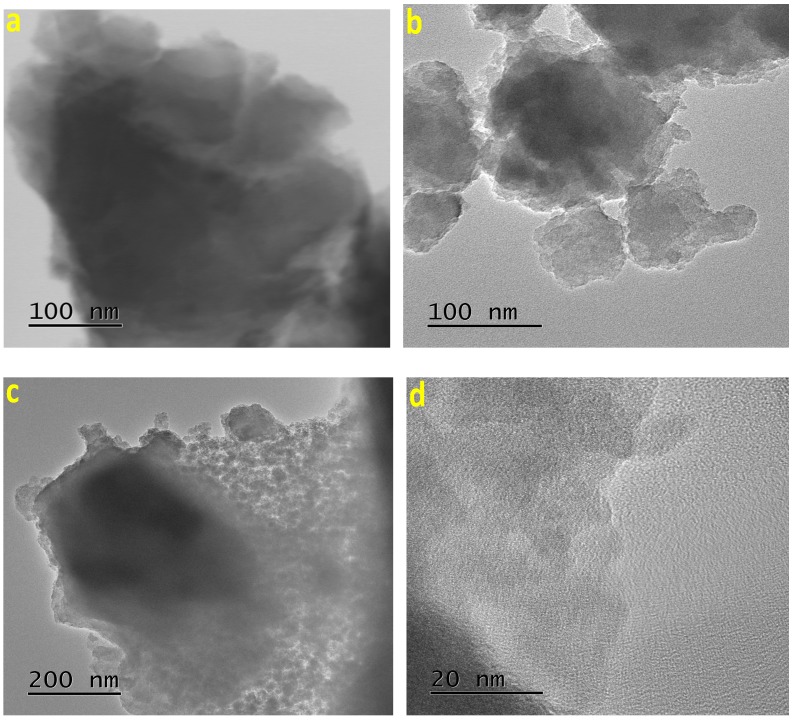
TEM images of the sample containing the CoTSPc species trapped inside the pores of: (**a**) ZrO_2_ (**a**,**b**) TiO_2_ networks. TEM images of TiO_2_ substrates containing trapped (**c**) NiTSPc (**c** or **d**) CuTSPc species.

In the TEM images of ZrO_2_ and TiO_2_ xerogels, including the MTSPc species physically trapped inside their pores, it is possible to clearly observe some other structural details (Figure 5 and Figure 6). For instance, in the sequence of images corresponding to the (OH)AlTSPc species trapped inside ZrO_2_ or TiO_2_ xerogels, it is possible to distinguish translucent particles with a relatively smooth surface, without evident sign of cavities or fractures (Figure 5a,c). However, at a higher magnification (Figure 5b,d), a different morphology and packing of focused particles can be observed. These differences could be associated to the internal porosity of both networks, which suggest the existence of smaller cavities in the TiO_2_ than in the ZrO_2_ network. Since both samples were synthesized from the same (OH)AlTSPc species, the textural differences can only be explained in terms of the different reactivity of the precursory alkoxide employed; *i.e.*, to the identity of the metallic cation present in the structure of the inorganic network.

In the case of the xerogels containing the CoTSPc species (Figure 6), it is possible to observe that the network is constituted by the aggregation of particles, whose clusters are larger in the ZrO_2_ than in the TiO_2_ matrix (Figure 6a,b). In a similar way, in the TEM images of TiO_2_ xerogels doped with the NiTSPc and CuTSPc species (Figure 6c,d), a network formed by the aggregation of particles of smaller sizes can be observed. On comparing the images taken at high magnifications (Figure 6a,b,d), it is possible to see differences in the sizes of the particles that form the network, which, apparently depend on the identity of the central cation of the MTSPc species. Furthermore, at relatively high amplification, the different opacity regions suggest the existence of very small cavities and necks connecting them, as shown in the image of the TiO_2_ xerogel with the CuTSPc species trapped inside.

**Figure 7:**
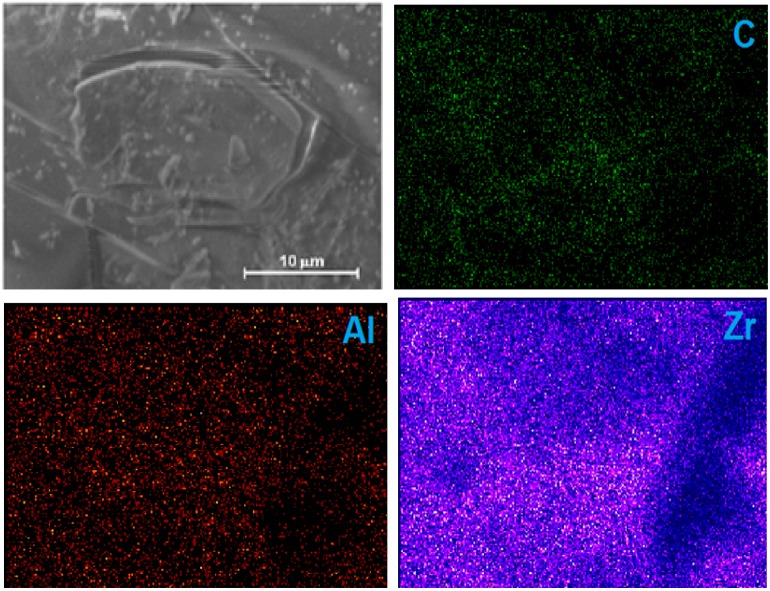
(**a**) SEM image of a ZrO_2_ xerogel with the (OH)AlTSPc species trapped inside the pores and treated at 125 °C, and (**b**) EDS mapping images of carbon C, aluminum (Al) and zirconium (Zr).

Due to their intrinsic nature, the SEM images of the xerogel sample (Figure 7) encapsulating the (OH)AlTSPc species show a smooth texture with no evidence (due to insufficient microscope resolution) of the existence of large pore cavities. However, the EDS element mapping reveals a homogeneous distribution of Zr, Al, and C. As an example, the EDS analysis of a sample doped with the (OH)AlTSPc species gives the following surface element wt % analysis: 0.37 of Al (1.00 mol), 1.31 N (6.82 mol), 16.33 C (99.14 mol), 41.4 (188.7 mol) of O, and 40.59 of Zr (32.45 mol). The percentage of Al and N can only be associated to the presence of aluminum phthalocyanine, with the molecular formula AlN_8_C_32_S_4_O_13_H_13_Na_4_. The carbon or oxygen excesses can be related to the same pthalocyanine complex as well as to the existence of remnant acetylacetone (acac), dimethylformamide (DMF) or propoxy groups (OC_3_H_7_). From the same detection source, the O/Zr molar ratio of about 5.5 mol O/mol Zr can be attributed to the presence of Zr-OH groups in the network. Furthermore, the above wt % analysis reveals that on the surface of the samples there exist 32 zirconium atoms per each (OH)AlTSPc molecule; most of these last atoms are forming part of the pore cavities that surround the macrocyclic trapped species. Similar percentages of carbon, oxygen and zirconium were determined from EDS after the corresponding microscopy analyses of the other hybrid samples.

**Figure 8:**
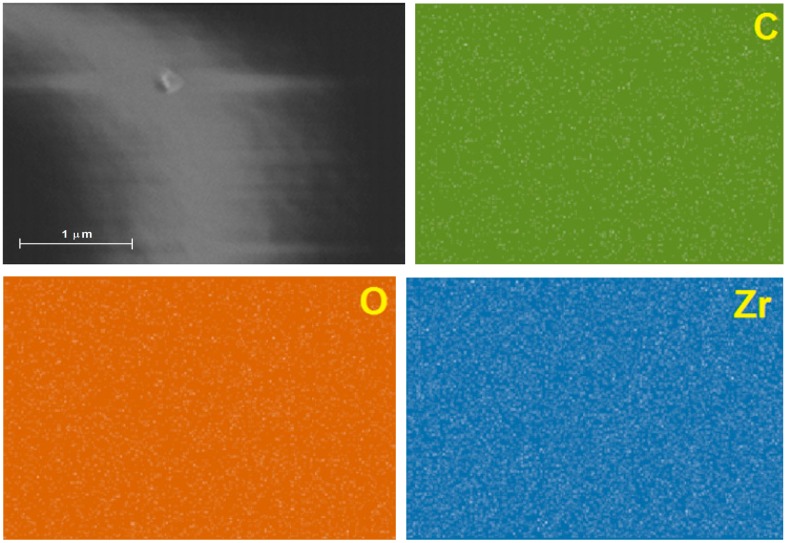
SEM images and EDS mapping images of a ZrO_2_ xerogel with the CoTSPc species trapped inside the pores.

In the same way, the SEM image of a ZrO_2_ xerogel, encapsulating the CoTSPc species, reveals a smooth surface without evident cavities (Figure 8), nevertheless, the microscope magnification employed is not enough to perceive small nanometric holes. Contrastingly, EDS mapping unveiled a homogeneous distribution of carbon, oxygen, and zirconium, whose weight percentages corresponded to 27.47%, 53.31%, and 18.17%, respectively. These data disclose the presence of organic materials related to the trapped macrocycle structure, as well as residual *propoxyde* groups and/or *acetylacetonate* remnants. The oxygen excess can be associated to the presence of numerous Zr-OH surface groups, which supply some hydrophilic character to the network. For the case of TiO_2_ xerogels, which include trapped MTSPc molecules, the SEM and EDS results were similar to those obtained for ZrO_2_ and therefore are not presented in this document.

### 2.4 Textural Characterization by N_2_ Sorption

**Figure 9:**
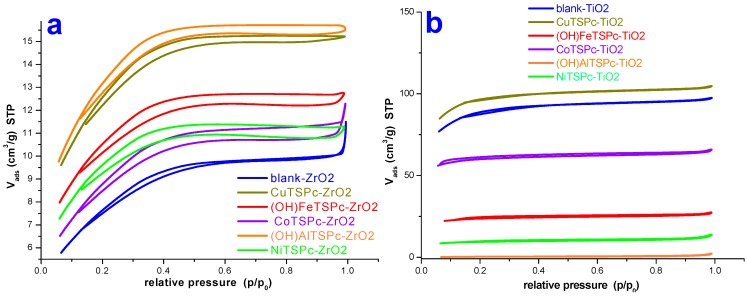
N2 sorption-desorption isotherms (at 76 K) on: (**a**) ZrO_2_ and (**b**) TiO_2_ xerogels containing the different MTSPc species trapped inside the pore network.

The N2 sorption-desorption isotherms measured at 76 K on the ZrO_2_ and TiO_2_ pristine networks, labeled as blank-ZrO_2_ (Figure 9a) and blank-TiO_2_ (Figure 9b), depicted similar isotherm pathways, with different N_2_ sorption capacities. Both isotherms can be classified as IUPAC Type I isotherms with H3 hysteresis cycles [61], which are representative of slit-like materials. The same isotherm characteristics occur for those samples entrapping the (OH)AlTSPc species while the other entrapped MTSPc species all depict similar shapes. In these types of networks, the condensation and evaporation of N2 molecules should take place relatively easy and without the irruption of intense cooperative phenomena or pore blocking effects. In the set of TiO_2_ samples, the hysteresis cycles were narrower than those corresponding to the ZrO_2_ samples; additionally, the N_2_ adsorption capacities of TiO_2_ samples resulted be higher than those of the ZrO_2_ samples. Furthermore, it is evident that the sorption capacity of each sample depends on the identity of the MTSPc compound trapped inside the porous network.

From the N2 isotherms, the specific surface areas and the average pore diameters (Φ) of each substrate were calculated (Table 1). This last parameter was obtained from application of the Non-Local Density Functional approach to the desorption boundary curve of the hysteresis loop of the isotherm, while assuming spherical cavities. In the case of the ZrO_2_ samples including or not MTSPc species trapped inside, the pore widths (Φ) and surface areas ranged in a narrow 2.2 to 2.4 nm interval and the surface area ranged from 28.1 to 48.3 m^2^/g. Contrastingly, in the case of the TiO_2_ samples, the average pore diameters (Φ) and surface area determined varied along a wider range of values, *i.e.*, 2.5 to 8.3 nm and 32 to 334 m^2^/g, respectively. For the pristine silica sample (blank-SiO_2_), the average pore diameter was 3.4 nm and the surface area accounted for 729 m^2^/g [32,34].

**Table 1:** Textural parameters evaluated from N_2_ sorption isotherms on ZrO_2_ or TiO_2_ xerogels containing trapped MTSPc species. The calculated values are compared against those previously reported for similar silica systems [32,34].

Network	Sample	Average Pore Diameter (Φ) (nm)	Specific Surface Area (m^2^/g)
ZrO_2_	with MTSPc (M = Fe, Co, Ni, Cu, Al)	2.2 to 2.4	28.1 to 48.3
blank-ZrO_2_		2.2	28.1
TiO_2_	with MTSPc (M = Fe, Co, Ni, Cu, Al)	2.5 to 8.3	32.0 to 334.0
blank-TiO_2_		2.5	308.0
SiO_2_	with MTSPc (M = Fe, Co, Ni, Cu, Al)	2.2 to 2.4 *	540.0 to 631.0 *
blank-SiO_2_		3.4 *	729.0 *

* Textural parameters taken from [32,34].

On comparing all the above data, the values of Φ = 2.2 to 2.4 nm and the surface areas from 540 to 631 m^2^/g, previously determined for silica systems [32,34], indicate that the identity of the trapped MTSPc species has an important effect over the textural characteristics of the final porous network. In the case of silica materials, the obtainment of smaller pore widths and surface areas imply that the encapsulation of macrocyclic species induces the creation of pores of smaller sizes, possibly due to the interaction between the silica network precursor and the sulfonate (-SO_3_Na) groups attached at the periphery of the MTSPc species. For the blank-ZrO_2_ and blank-TiO_2_ substrates the average pore diameters and surface areas were of the same order of magnitude as those determined for the samples synthesized in the presence of MTSPc species, thus suggesting a weak effect of the macrocyclic species over the formation of cavities in the network. However, for the TiO_2_ set of samples, both parameters are larger; since all samples were synthesized by employing the same precursory molar mixtures, but different MTSPc species, the only reason of these differences is the cation present in those macrocyclic complexes, which apparently strongly affects the size of the pores in the final network.

Based on all the above reported data, the process of generation of cavities created from alkoxides can be described as follows: after a rapid hydrolysis and polycondensation reactions, metaloxane bridges (M-O-M) start to be formed. The high reactivity of Zr and Ti alkoxides is accountable for the creation of very small nucleation centers (primary particles). In the absence of doping species, a large number of small particles creates compact aggregates, which inhibit the free access of larger molecules into their interior as consequence of the very small pore throats that are interconnecting neighboring cavities. The porous structure can be modified from depicting a Type I to a Type IV isotherm when treating the xerogel at higher annealing temperatures. *i.e.*, the porous structure is opened up by the evolution of volatile materials still remaining in the xerogel structure (e.g., *acac*) as the hybrid material is treated at higher temperatures. Other alternative consists in use of relatively large templating species, such as the tetrapyrrole macrocyclic molecules, in the xerogel network. The results showed herein, suggest the interactions between network precursors (primary or higher aggregates) and some the most polar regions of the tetrapyrrolic macrocycle complex. This induces the formation of cavities, whose size depends on the structure of the macrocycle and the identity of the central cation contained in it. Wider interconnections are established between neighboring pore cavities as consequence of the modulating action of the trapped molecules.

Furthermore, by considering an approximate size of the MTSPc species of about 1.8–2.0 nm, the average pore diameters existing in the three type of networks, herein analyzed, resulted of similar dimensions, which easily allows the trapping of one molecule of MTSPc or as most probably occurs; that pore cavity formation takes place around the solvated MTSPc species and that the –SO_3_Na groups interact strongly with the precursory network species and having the M-OH surface groups attached to the pore walls. For example, the surface areas ranged from 32.4 to 48.3 m^2^/g and resulted to be slightly higher than that of the pristine ZrO_2_ sample (28.1 m^2^/g). If this estimation is accurate, the most drastic effect over the surface area of xerogels containing trapped MTSPc species can be better understood.

### 2.5 Covalent Bonding of Cobalt Tetrakis-para-Carboxyphenyl-porphyrin Inside ZrO_2_ or TiO_2_ Xerogels

**Scheme 1:**
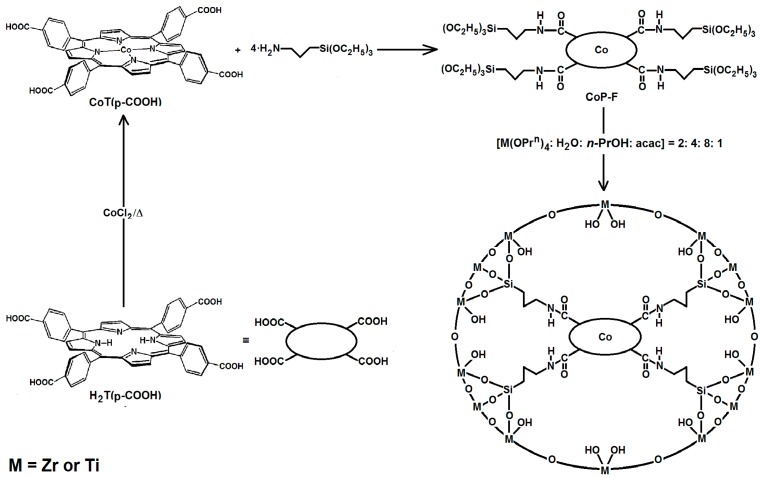
Reaction scheme for the covalent union of the cobalt complex of tetrakis-(*p-*carboxyphenyl)-porphyrin, (H_2_T(*p-*COOH)PP or CoT(*p-*COOH)PP) the free base to ZrO_2_ or TIO_2_ xerogel networks.

In order to extend the developed methodology for fixing different tetrapyrrole macrocycles or other chemical species inside xerogel pore networks, some exploratory experiments were conducted by choosing the cobalt complex of tetrakis*-para*-carboxyphenyl-porphyrin, CoT(*p-COOH*)PP, as an insertion probe molecule. Since no commercial functionalized zirconium or titanium alkoxides exist on the market, silicon alkoxides such as 3-aminopropyltriethoxysilane (APTES) and 3-isocyanato-propyltriethoxysilane (IPTES) can be tested as bridging compounds. When proceeding in this way, a first step consists in establishing covalent unions between the carboxyl groups (-COOH) of the porphyrin and amine groups (-NH_2_) of APTES to render the respective CoP-F precursor (Scheme 1 in Experimental Section), which was monitored through FTIR spectroscopy, as it has been previously reported [39,40]. In a second step, the synthesized CoP-F precursor was redissolved in 1-propanol (*n-*PrOH) and combined with the adequate gelling mixture of M(OPr)_4_:H_2_O:*n-*PrOH:acac, in order to generate ZrO_2_ or TiO_2_ matrixes, as it is required for performing the physical entrapping of the macrocycle inside the xerogel network.

**Figure 10:**
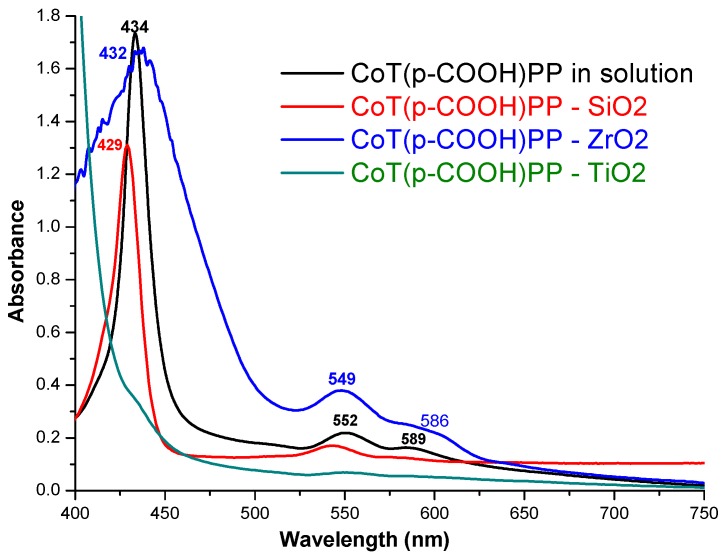
UV-Vis spectra of CoT(*p-*COOH)PP species in solution or when chemically bonded to SiO_2_, ZrO_2_ and TiO_2_ pore networks.

The UV-Vis spectrum of the precursory CoT(*p-*COOH)PP species in solution shows the characteristic signals of a metalloporphyrin with a di- or trivalent cation, as confirmed by the Soret band (B) at 434 nm and the two Q bands at 552 nm (Q_III_) and 589 nm (Q_II_) (Figure 10), respectively. Experimental results previously reported, concerning the bonding of the CoT(*p-*COOH)PP species inside the pores of a silica network, reveal a Soret band at 429 nm together with Q_III_ and Q_II_ bands at 549 and 588 nm, respectively [39]. The UV-Vis spectra of the CoT(*p-COOH*)PP species bonded to the different inorganic networks reported herein, show a similar pathway of the Soret and Q_III_ and Q_II_ bands at 436 nm (B), 549, and 586 nm for the ZrO_2_ xerogel, respectively [53]. In the case of the TiO_2_ xerogel these signals were observed at 552 nm and 588 nm and the Soret band appeared as a shoulder at 433 nm. In both cases, the permanence of the shape and position of the bands suggest that the bonded macrocyclic species remain attached in monomeric and stable form inside the pores of the ZrO_2_ and TiO_2_ networks. In the case of the ZrO_2_ xerogel, the existence of a wide Soret band can be due to the superposition of this signal with the electronic transitions of the ZrO_2_ network, or it can also be attributed to the interaction of the porphyrinic complex with the surface Zr-OH groups located on the pore walls. In the case of TiO_2_ xerogels, the Soret band appears overlapped with an intense band associated to remnant acetylacetone that is coordinated to titanium groups in the xerogel network. However, the absence of traces of porphyrin complexes in the solvents employed to wash the xerogels, together with the distinctive UV-Vis signal pathway, demonstrate that, in all the synthesized systems, the porphyrinic complex has been unequivocally bonded to the network in monomeric and chemically stable forms.

This last observation suggests the possibility of applying the developed methodology to the bonding of the free bases or to the respective metal complexes of other synthetic or natural tetrapyrrolic macrocycles. It is well known than acid medium could make the porphyrinic complex to lose its central metal cation then forming a dicationic porphyrin, H_4_P^4+^, and making evident the demetallation and protonation of the complex. When this last event takes place, the purple or reddish porphyrin solution turns into green. In the UV-vis spectrum, these changes are revealed by a Soret band shifted to higher wavelengths and to the substitution of the Q_II_ and Q_III_ bands by a more intense Q_I_ band at around 650 nm. The absence of these evidence in the respective UV-Vis spectra, suggest that no demetallation or protonation of the CoT(*p-*COOH)PP species takes place when this species is bonded and trapped inside ZrO_2_, TiO_2_ or SiO_2_ networks.

**Figure 11:**
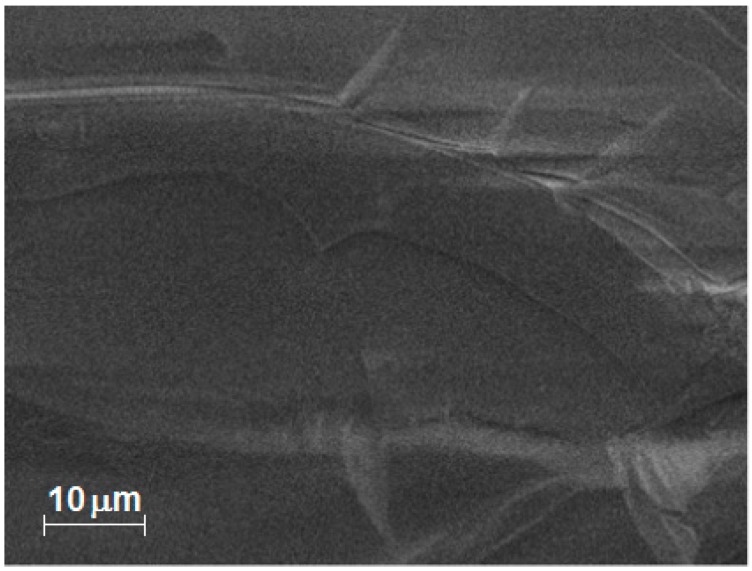
SEM image of a ZrO_2_ xerogel with CoT(*p-*COOH)PP species trapped inside the pores and thermally treated at 125 °C.

SEM images of ZrO_2_ xerogels, with the CoT(*p-*COOH)PP species bonded to the pore network reveal a smooth surface free of great cavities or cracks (Figure 11). The associated EDS mapping reveals homogeneous distributions of zirconium, oxygen and carbon, which percentage masses of 20.39%, 53.12%, and 25.05%, respectively. Again, the high mass percentage determined for oxygen can be due to the network formation, in which not all oxygen atoms are linked to two zirconium atoms, but some of them remain as chemisorbed water or M-OH groups. Furthermore, the carbon excess can be attributed principally, to the presence of porphyrin macrocycles (H_2_T(*p-*COOH)PP = C_48_H_30_N_4_O_8_), or to propylamide bridges (-CH_2_-CH_2_-CH_2_-NH-CO-), while a lesser amount stays as remnant acetylacetone or propoxide groups.

**Figure 12:**
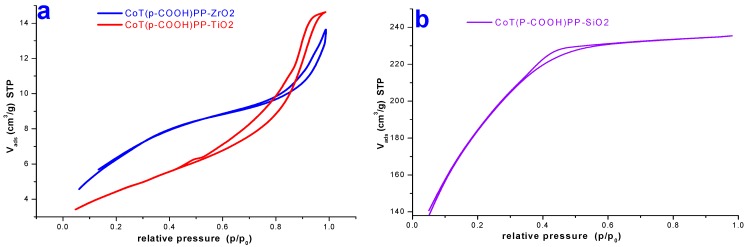
N2 sorption-desorption isotherms (at 76 K), measured after thermal treatment at 125 °C, on: (**a**) ZrO_2_ and (**b**) TiO_2_ xerogels containing the CoT(*p-*COOH)PP species bonded to the pore walls.

The N2 sorption isotherms at 76 K related to ZrO_2_ and TiO_2_ substrates with cobalt porphyrin bonded to the pore walls could be classified as IUPAC Type IV isotherms with H3 hysteresis cycles, characteristic of mesoporous solids in which the pore morphology can be due to the association of slit-like aggregates of particles (Figure 12). In the case of the SiO_2_ sample, the isotherm resulted to be very different and can be classified as an Type I with a narrow hysteresis cycle, which can be linked to a mesoporous network made of relatively small void sizes (this being the reason of the narrow hysteresis loop); it is important to remark that the specific surface area is larger than those associated to the ZrO_2_ and TiO_2_ specimens. Besides, in a previous paragraph, it was mentioned that the water reabsorption process was more intense and faster in the silica xerogel, if compared to the other metal oxide substrates. The NLDFT average pore diameters, (Φ), were calculated from the N_2_ desorption curves (assuming spherical pore cavities) and their values resulted to be of 3.4 nm with a specific BET surface area of 23.2 m^2^/g for the ZrO_2_ sample and 3.4 nm and 161 m^2^/g in the case of the TiO_2_ sample. In the analogous silica system, the average pore width was 3.1 nm and the BET surface area of 553.8 m^2^/g [39,40]. It is well known that specific surface areas of around 100 m^2^/g are typical of mesoporous ZrO_2_ samples. The obtainment of a low surface area for the ZrO_2_ xerogel can be caused by the composition of the precursory molar mixture that was used, since the obtained value resulted to be similar to the value of 28.1 m^2^/g determined for the pristine network (blank-ZrO_2_). In the case of the TiO_2_ xerogel with the porphyrin bonded to the network, the reduction in the surface area can only be attributed to the effect of the precursory species, CoP-F over the cavity growth.

As it was mentioned previously, for xerogels containing trapped MTSPc species, there only exists a slight difference among the pore diameters determined for the samples including or not trapped macrocyclic species. However, it is evident that, if compared to the respective references or blank samples, the average pore diameters resulted larger in systems containing the CoT(*p-*COOH)PP species bonded to the network. The very similar values of pore widths determined for the different networks can be due to the templating effect exercised by the use of the same CoP-F precursory species (see the Experimental Section).

**Figure 13:**
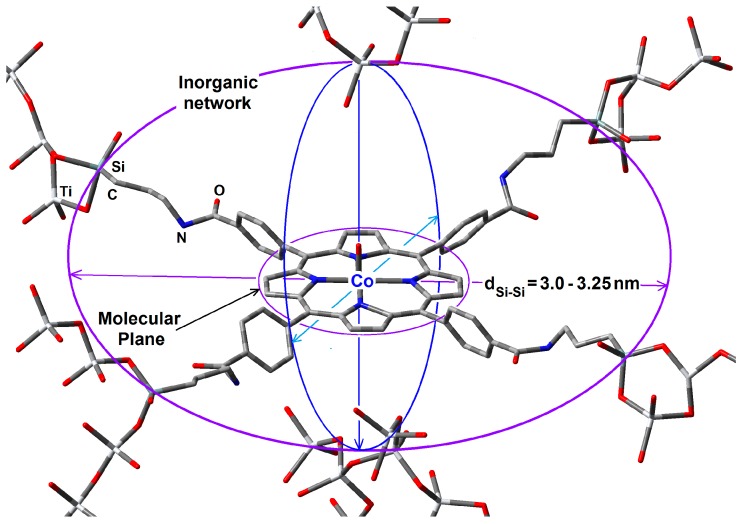
Hypothetical situation of the CoT(*p-*COOH)PP species bonded to the TiO_2_ pore network by means of silicon precursory alkoxides, which restrains the growing of the network at the periphery of the trapped macrocycle without inhibiting the approaching of gelling particles at both sides of the molecular plane. Hydrogen atoms are omitted for simplicity.

These similar results are, however, relatively obvious bearing in mind that the distance between the two opposite silicon atoms (d_Si-Si_) in such precursory molecules, is about 3.0 to 3.25 nm. In all the synthesized systems presented herein, the hydrolysable and condensable silicon atoms existing in the precursory species function as nucleation points that create the cavities, which start growing around the macrocyclic species. Due to the finite separation existing between the hydrolysable silicon atoms, the size and shape of the network in formation depend on the position of these groups around the macrocycle molecule. In the particular case of the CoT(*p-*COOH)PP species, the hydrolysable groups are localized at the periphery of the macrocycle in the proximity of the molecular plane (Figure 13). In this way, the connection with the pore walls occurs from those four silicon atoms, whose separation determines the width of the cavity. However, the approximation of the precursory network particles is not very much inhibited at both sides of the molecular plane, thus causing the formation of an ellipsoidal pore. As it was mentioned before, pore diameters were evaluated by assuming spherical cavities; however, as it was pointed out, the interactions of the macrocycle with the precursory network species could induce the formation of ellipsoidal cavities. The above results suggest that, the specific surface area is the result of the precursory molar mixture employed and can be imputable to the intrinsic nature of the alkoxide chosen; however, the pore size widths can be controlled some way through the use of templating species, such as the tetrapyrrolic macrocycles employed herein.

In the particular case of the abovementioned systems, the slight difference of average pore diameters between ZrO_2_, TiO_2_ and the silica samples could be caused by a more drastic contraction of the silica gel after the shrinkage step, as consequence of the use of more water added to the precursory molar mixture employed for the reaction. On the other hand, the higher pore diameter values determined for ZrO_2_ and TiO_2_ samples can attributed to a faster consolidation and formation of more rigid networks combined with a lower shrinkage of the resultant gels.

From all the evidence, it is possible to affirm that preservation of the inherent physicochemical properties of trapped or bonded physicochemical active molecules inside different metal oxide networks could render very interesting hybrid materials suitable for important application in diverse strategic and technological areas as optics, catalysis, compound sensing, and medicine.

## 3. Experimental Section

### 3.1. General Information

UV-visible and near infrared (NIR) spectra were obtained via a Cary-Varian 500 E spectrophotometer (Varian Optical Spectroscopy Instruments, Mulgrave, Victoria, Australia); infrared spectra (FTIR) were measured on a Perkin-Elmer GX FTIR instrument (Perkin-Elmer Inc., Waltham, MA, USA). Nuclear Magnetic Resonance spectra were recorded on a Bruker Advance +300 spectrometer (Bruker BioSpin Corporation, Billerica, MA, USA). TEM images were taken with a JEOL 7600F microscope (JEOL Ltd. Akishima, Tokyo, Japan) coupled to an Oxford Instruments INCA EDS detector (Oxford Instruments, Concord, MA, USA). N2 adsorption-desorption isotherms were measured on a Micromeritics ASAP 2020 instrument (Micromeritics Instrument Corporation, Norcross, GA, USA) at 76 K (boiling point of N2 at the México City’s 2250 m altitude). Pore size distributions (PSD), inherent to the networks containing the trapped macrocyclic species, were calculated by the NLDFT method applied to the boundary desorption curve of the N2 isotherms [62,63] and assuming spherical pore cavities.

### 3.2. Synthesis of Tetrapyrrolic Macrocycles

Metal tetrasulphophthalocyanine complexe2, MTSPc (where M ≡ Fe, Co, Ni, Cu, and Al), were synthesized and purified by the Weber and Busch method [64]. The free (unmetallated) base of tetrakis-(*para-carboxy*phenyl)porphyrin, H_2_T(*p-*COOH)PP, was synthesized and purified through the Rothemund [65] reaction, while following the Adler methodology [66]. Cobalt tetra-(*para*-carboxy-phenyl)porphyrin complex, CoT(*p-COOH*)PP, was synthesized by refluxing a 1:1 molar mixture of cobalt chloride, CoCl_3_, and the respective H_2_T(*p-*COOH)PP porphyrin free base. The ensuing compound was purified through silica gel chromatography. All the resultant macrocyclic compounds were characterized by UV-Vis, FTIR, elemental analysis, and NMR.

### 3.3. Macrocycle Trapping inside ZrO_2_ or TiO_2_Pore Networks

ZrO_2_ and TiO_2_ pore networks were prepared from the respective zirconium or titanium tetrapropoxides, (Zr(OPr^n^)_4_ or Ti(OPr^n^)_4_), dissolved in 1-propanol (*n-*PrOH), whose reactivity was regulated through previous coordination with acetylacetone (acac). μ*-oxo-*hydroxyaluminum (III) tetrasulfophthalocyanine, (OH)AlTSPc, was used as a probe species to establish the following Zr(OPr^n^)_4_:H_2_O:*n-*PrOH:*acac* molar mixture sequence of 2:2.5:7.5:1 that guaranteed the synthesis of translucent and monolithic ZrO_2_ xerogels [53]. Besides, the resultant xerogels should have the convenient surface groups as to allow the physical trapping or covalent bonding of macrocyclic species, in monomeric and stable forms, inside the pore network. Similarly, monolithic xerogels of translucent TiO_2_ could be synthesized from the following Ti(OPr^n^)4:H2O:*n-*PrOH:acac molar mixture ratio of 5.3:5:7:1. This recipe avoided the possibility of fractures arising during the drying step while inducing the creation of hard monoliths; a small amount of dimethylformamide (DMF) was also added to regulate the sol-gel hydrolysis reaction.

As an example, the Zr gelling mixture was prepared from a solution of acetylacetone (0.42 mL) in *n-*PrOH (1.2 mL), which was combined with a 4.04 × 10^−4^ M MTSPc solution in water (0.2 mL), together with acac (0.1 mL) and DMF (0.083 mL) included as an aggregation inhibiting agent. This mixture was poured dropwise into a solution of Zr(OPr^n^)_4_ in 1-propanol (3.8 mL, 70% *v*/*v*). By respecting the above optimal composition sequence of molar ratios, it was possible to reach an adequate inclusion of (OH)AlTSPc probe molecules within the pore structure of ZrO_2_ xerogels. In summary, it was possible to physically entrapping different MTSP complexes, within ZrO_2_ pore networks, all of them in monomeric and stable forms. A similar procedure, but now employing the appropriate amounts of reactants shown in Table 1, was followed to synthesize translucent TiO_2_ xerogels.

### 3.4. Synthesis of ZrO_2_ and TiO_2_ Monolithic Translucent Xerogels 

The syntheses of ZrO_2_ and TiO_2_ translucent xerogels of entrapping either porphyrin free bases or their respective cobalt complexes (*i.e.*, H_2_T(*p-*COOH)PP and CoT(*p-*COOH)PP), covalently bonded to the pore walls of the xerogel network, require firstly obtaining the respective H_2_P-F and CoP-F hydrolysable precursor species. These precursors can be obtained from the reaction between the carboxyl substituent groups of the macrocyclic species and the amine groups of 3-aminopropyl-triethoxysilane (APTES) acting as a bridging species (Scheme 1). In a second step, these precursors were covalently bonded to the pore walls of the Zr and Ti networks through hydrolysis of the ethoxy groups of APTES and their subsequent polycondensation with similar hydrolyzed species proceeding from the respective Zr and Ti propoxides.

### 3.5. Synthesis of Xerogels Containing CoT(P-COOH)PP Species Covalently Bonded to the Pore Walls

In order to perform this linking action, the alkoxide precursor (0.3125 mL) was combined with H_2_O (0.2 mL) and mixed with the necessary volumes of Zr(OPr^n^)_4_, *n*-PrOH, and Hacac compounds, according to the same molar ratio series established before [53]. After gellification and conclusion of the shrinkage step of the solid samples, these substrates were dried for a week at room temperature; afterwards, for 1 day at 75 °C and one more day at 125 °C. Finally, the samples were washed with water, ethanol, propanol, acetone, and chloroform to eliminate traces of unbound macrocyclic compound.

By using the same molar ratios, some reference samples were synthesized but with no trapped macrocycle molecules (Table 2). All samples were next characterized by UV-Vis (employing the blank-ZrO_2_ or blank-TiO_2_ samples as background), medium and near infrared spectroscopies (FTIR and NIR), as well as by X-ray powder diffraction, N2 sorption at 76 K, TEM, and EDS.

**Table 2:** Composition of the gellifying molar mixtures, as determined through the use of the (OH)AlTSPc species, employed as a probe for the synthesis of ZrO_2_, TiO_2_ and SiO_2_ translucent monolithic xerogels.

Sample	Alkoxide/Volume (mL)	*n*-PrOH (mL)	acac (mL)	DMF (mL)	HCl 0.248 M (mL)	(OH)AlTSPc (mL)	[Macrocycle] * mol/L
ZrO_2_	Zr(OPr^n^)_4_/3.8 mL	1.2	0.42	0.083		0.2	4.04 × 10^−4^
blank-ZrO_2_	Zr(OPr^n^)_4_/3.8 mL	1.2	0.42			0.2	
TiO_2_	Ti(OPr^n^)_4_/3.0 mL	1.0	0.2	0.3		0.2	4.04 × 10^−4^
blankTiO_2_	Ti(OPr^n^)_4_/3.0 mL	1.0	0.2			0.2	
SiO_2_	Si(OEt)_4_ = TEOS	9.8		0.5	10	5.0	10^−3^−10^−7^
blank-SiO_2_	Si(OEt)_4_ = TEOS	9.8			10	5.0	

* [Macrocycle] = Macrocycle Concentration.

## 4. Conclusions

Aluminium tetrasulfophthalocyanine, (OH)AlTSPc, was used as a probe to find the appropriate molar ratio sequence of Zr(OPr^n^)_4_:H_2_O:HOPr^n^:acac that allowed the synthesis of translucent, monolithic ZrO_2_ or TiO_2_ xerogels, with the macrocyclic species trapped in chemically stable and monomeric form. By using this molar mixture, it was possible to trap some other metal tetrasulfophthalocyanines, MTSPc (M = Fe, Co, Ni, Al and Cu) inside ZrO_2_ and TiO_2_ pore networks. Even if the CuTSPc complex remained trapped in monomeric form inside a ZrO_2_ network, this same species formed aggregates inside the pores of SiO_2_ andTiO_2_ networks. 

This result, together with characterization by near infrared analysis, confirmed a weak hydrophilic character of the surface, as well as the existence of a smaller amount of ZrOH surface groups, in contrast to what happens in SiO_2_ xerogel trapping systems. The same methodology was successfully applied for covalently bonding the CoT(*p-*COOH)PP compound onto the pore walls of ZrO_2_, through the bridging action of amine-functionalized silicon alkoxides. The average pore widths determined for these samples were very similar to those found for analogous silica systems, thus indicating that the pore cavity created around the macrocycle species depends on the size of this very molecule. The results herein obtained demonstrate that is possible to successfully trapping synthetic tetrapyrrole macrocyclic species, such as phthalocyanines, porphyrins or parent natural species as for instance chlorophylls and blood’s heme group, inside the pores of ZrO_2_ networks. Through the developed methodology, the transcendental physicochemical properties that free tetrapyrrole macrocycles display in solution, can be even better displayed or tuned up inside new hybrid solid systems, which could be fruitfully exploited in diverse technological fields.
